# Asthma and Technology in Emerging African American Adults (The ATHENA Project): Protocol for a Trial Using the Multiphase Optimization Strategy Framework

**DOI:** 10.2196/37946

**Published:** 2022-05-10

**Authors:** Alan Baptist, Wanda Gibson-Scipio, April Idalski Carcone, Samiran Ghosh, Angela J Jacques-Tiura, Amy Hall, Karen Kolmodin MacDonell

**Affiliations:** 1 Department of Internal Medicine University of Michigan Ann Arbor, MI United States; 2 Department of Health Behavior and Health Education University of Michigan Ann Arbor, MI United States; 3 College of Nursing Wayne State University Detroit, MI United States; 4 Department of Family Medicine and Public Health Sciences School of Medicine Wayne State University Detroit, MI United States

**Keywords:** African American emerging adults, asthma management, mHealth, mobile health, motivational interviewing, asthma control, physical activity

## Abstract

**Background:**

Asthma causes substantial morbidity and mortality in the United States, particularly among African American emerging adults (AAEAs; aged 18-30 years), but very few asthma programs have targeted this population. Interventions that provide education and address underlying motivation for managing asthma may be the most effective. However, intensive face-to-face interventions are often difficult to implement in this population.

**Objective:**

The purpose of this study is to develop an effective mobile asthma management intervention to improve control among AAEAs.

**Methods:**

We will assess the ability of multiple technologic components to assist and improve traditional asthma education. The first component is the Motivational Enhancement System for asthma management. It is a mobile 4-session intervention using supported self-regulation and motivational interviewing. Personalized content is based on each participant’s activity level, daily experiences, and goals. The second component is supportive accountability. It is administered by asthma nurses using targeted mobile support (Skype/voice calls) to provide education, promote self-efficacy, and overcome barriers through a motivational interviewing–based framework. The third component is SMS text messaging. It provides reminders for asthma education, medication adherence, and physical activity. The fourth component is physical activity tracking. It uses wearable technology to help meet user-defined physical activity goals. Using a multiphase optimization strategy (MOST) framework, we will test intervention components and combinations of components to identify the most effective mobile intervention. The MOST framework is an innovative, and cost- and time-effective framework that uses engineering principles to produce effective behavioral interventions. We will conduct a component selection experiment using a factorial research design to build an intervention that has been optimized for maximum efficacy, using a clinically significant improvement in asthma. Participants (N=180) will be randomized to 1 of 6 intervention arms. Participants will be recruited from multiple sites of the American Lung Association-Airway Clinical Research Centers network and ambulatory care clinics at the Detroit Medical Center. Data collections will occur at baseline, and 3, 6, and 12 months.

**Results:**

At study completion, we will have an empirically supported optimized mobile asthma management intervention to improve asthma control for AAEAs. We hypothesize that postintervention (3, 6, and 12 months), participants with uncontrolled asthma will show a clinically significant improvement in asthma control. We also hypothesize that improvements in asthma management behaviors (including physical activity), quality of life, symptoms, adherence, and exacerbation (secondary outcomes) will be observed.

**Conclusions:**

AAEAs are disproportionately impacted by asthma, but have been underrepresented in research. Mobile asthma management interventions may help improve asthma control and allow people to live healthier lives. During this project, we will use an innovative strategy to develop an optimized mobile asthma management intervention using the most effective combination of nurse-delivered asthma education, a smartphone app, and text messaging.

**International Registered Report Identifier (IRRID):**

PRR1-10.2196/37946

## Introduction

### Background

Health disparities are evident in many conditions, including cancer, diabetes, and cardiovascular disease. One condition with a significant inequality in health outcomes is asthma. Recent prevalence rates for asthma in the United States show that 7.9% of the adult population has asthma, and this rate is 10.1% among African Americans or Black people. Unfortunately, severity and disease control disparities are significantly worse than the prevalence rates would suggest. For example, the asthma mortality rates are nearly 3 times higher among non-Hispanic African Americans or Black people than among non-Hispanic Whites (22.7 vs 8.1 per million) [1]. Additionally, hospitalization rates are 4 to 5 times higher among African Americans than among Whites [2,3]. Many factors may contribute to these disparities, including both environmental and underlying genetic influences. Urban areas, which often have a predominance of African American residents, are heavily concentrated with risk factors associated with asthma, such as air pollution [4], cockroaches [5], dust mites [6], poor diet [7], poverty [8], stress [9], and violence [10]. Studies have compared African American patients and White patients, and have found that African American patients visit an asthma specialist less often, receive less asthma education, and use an inhaled corticosteroid for persistent asthma less frequently [8,11-13].

### African American Emerging Adults With Asthma

This project will focus on African American emerging adults (AAEAs) between the ages of 18 and 30 years, a developmental period largely neglected in the research literature but known to have some of the worst asthma outcomes along with significant health disparities. While significant research has focused on outcomes in adolescence, most markers of asthma control (mortality, quality of life, and health care utilization) are even worse in emerging adults than in adolescents [14,15]. Part of the reason may be that emerging adulthood represents a period of dramatic change for an individual, with identity exploration and self-focus, increased independence and risk-taking, and decreased parental oversight [16,17]. For the first time, emerging adults are responsible for their own health care, finances, education, and employment. Compounding this problem, many pediatricians stop caring for patients once they turn 18 years. Adult practitioners may not be fully trained or equipped to deal with the unique circumstances of emerging adults transitioning out of adolescence [18] or may rely on the “developmental continuity myth,” that is, the fallacy of applying adult treatments to youth, with little alteration or changes to the interventions [19]. Successful interventions to improve health outcomes must be developed to meet emerging adults’ distinctive developmental needs. 

Literature on interventions to improve asthma management in emerging adults is nearly nonexistent [20-22]. Further, African Americans of all ages are underrepresented in mobile health (mHealth) and eHealth studies [23]. Results of studies with racial/ethnic minority adults suggest that culturally appropriate programs that target specific aspects of asthma management (eg, adherence and physical activity) have largely been successful [24]. Thus, interventions that similarly target AAEAs at the highest risk for poor asthma management and health outcomes may have promise for improving morbidity and mortality rates. Interventions must consider the target group’s distinctive cultural and developmental needs and challenges. This project tests a developmentally and culturally appropriate intervention for AAEAs with persistent asthma.

### Call for Technology-Based Mobile Interventions

There are multiple barriers to intervention delivery, including buy-in at multiple levels (eg, department and community), personnel turnover, and reproducibility. Emerging adults may also be unlikely to complete intensive in-person interventions [25]. Technology-based interventions cannot replicate important human elements of interventions, but they offer advantages in terms of reach, cost, anonymity, and scalability. While there are over 500 asthma-related apps in the Apple Store and Google Play Store, a review in 2019 found that less than 10 have actually been evaluated in clinical trials [26]. Most of these evaluations were conducted with adolescents, were focused on caregivers, or were implemented in school settings. Attrition in eHealth interventions is also common [27], and very few mHealth interventions have been grounded in behavior change theory. The use of SMS text message reminders can improve self-management in people of all ages with asthma [28,29], though the magnitude of improvement tends to be small. Mobile interventions have rarely examined the integration of remote nursing support with a theory-based program. The synergism of such an approach has potential additive effects that urgently require further exploration. Finally, mHealth interventions can be designed to address the concerns and barriers faced by racial/ethnic minority groups or those of low socioeconomic status, as the challenges and goals are often different among such populations [26]. The proposed project will utilize an innovative and cost-efficient framework to identify the most effective combination of intervention components to improve asthma control in AAEAs.

### Theoretical Model

**Figure 1:**
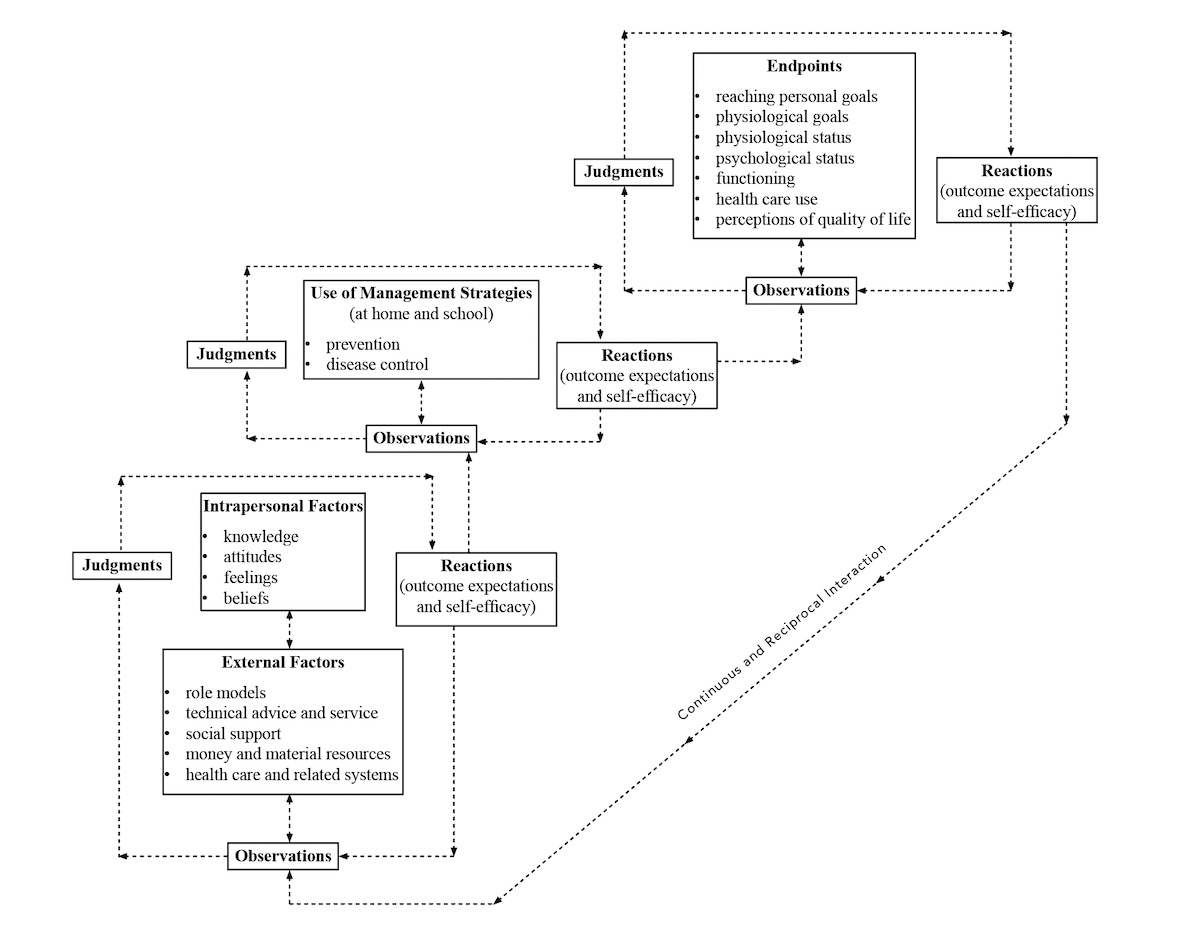
Self-regulation processes in disease prevention and management.

**Figure 2:**
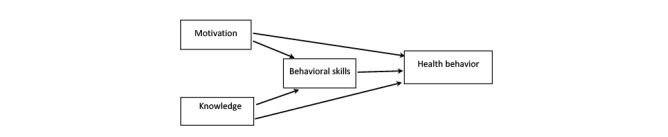
Information-Motivation-Behavioral Skills model.

Nearly every guideline notes that asthma education plays a critical role in disease management [30]. Educational strategies based on behavioral theories of change, such as motivational interviewing (MI), are more likely to be effective than strategies that simply provide information. MI is a collaborative goal-oriented style of communication with particular attention to the language of change. It is designed to strengthen intrinsic motivation for and commitment to a goal by eliciting and exploring the person’s own reasons for change within an atmosphere of acceptance and compassion. Research on the mechanisms of effect in MI has concluded that patient “change talk” (ie, statements about their own desire, ability, reasons, or commitment to change) consistently predicts actual behavior change. Several behavior change theories are consistent with MI. First, the Self-Regulation Theory (SRT) has core principles hypothesizing that multiple factors interact to influence behavior. The most influential way in which a person develops expectations and solidifies a behavioral change is through personal experience, which underlies improvement through self-regulation [31]. Zimmerman described self-regulation as a process of the following 3 phases: self-observation, self-judgment, and self-reaction [32], and Zimmerman and Clark adapted these subprocesses for asthma [33]. Through the self-regulatory process, the person is able to observe and learn from experience and determine ways for changing behavior (Figure 1). Second, the Information-Motivation-Behavioral Skills (IMB) model [34] posits that behavior change results from the joint function of the following 3 components: accurate information about risk behaviors (eg, not managing asthma) or their replacement behaviors (eg, benefits of following an asthma management regimen), the motivation to change behavior, and the perceived behavioral skills necessary to perform the behavior (eg, self-efficacy) (Figure 2). A powerful attribute of the IMB model is its ability to improve the self-efficacy needed for behavior change. For the intervention, we have combined the most powerful components of these complementary theories to build an asthma management intervention that not only targets asthma education, but also addresses underlying motivation and provides social support for change.

### Study Aims

The purpose of this study is to develop an effective mHealth intervention to improve asthma management and asthma control among AAEAs. We have developed and tested technology-based theoretically grounded asthma self-management interventions for AAEAs that will be refined and evaluated as components of a resulting mobile asthma management intervention. These intervention components are based on the SRT or IMB model, and utilize MI. We will evaluate 4 intervention components for synergistic effects. The first component is the Motivational Enhancement System (MES) for asthma management. The MES is a mobile 4-session MI intervention using supported self-regulation to enhance motivation for asthma management and asthma knowledge. Personalized content is chosen by a computer algorithm, and is based on each participant’s self-regulation stage, activity level, daily experiences, and goals. The second component is supportive accountability (SA). SA is administered by asthma nurses using targeted mobile support (Skype/voice calls) to provide education, promote self-efficacy, and overcome barriers through an MI-based framework. The third component is SMS text messaging (TXT). It provides asthma management education. The fourth component is physical activity tracking (PAT). PAT uses wearable technology (eg, a smart watch) for meeting user-defined activity goals as part of the asthma management regimen.

Participants will be recruited from multiple sites of the American Lung Association-Airway Clinical Research Centers network and clinics at Wayne State University/Detroit Medical Center and the University of Michigan. Participants (N=180) with uncontrolled persistent asthma will be randomized to 1 of 6 intervention arms consisting of combinations of the 4 components through a multiphase optimization strategy (MOST) design. The MOST design is an innovative, and cost- and time-effective experimental design that utilizes engineering principles to test behavioral interventions. The MOST design uses an optimization criterion to make decisions and refine the efficacy of each of the intervention components. We will adopt clinically significant improvements in asthma control (defined as a change of ≥3 on the Asthma Control Test [ACT]) as an optimization criterion in a component selection experiment using a randomized factorial design. This experiment is equivalent to conducting multiple pilot randomized clinical trials to evaluate the efficacy of each component, yet uses only a fraction of the sample size, resources, and time. At the completion of this study, we will have developed an empirically supported mobile asthma management intervention to improve asthma control for high-risk AAEAs.

## Methods

### Study Participants and Setting

Phase 2 consists of a clinical trial study. The study will include 180 AAEAs (30 per arm; N=150 + 20% attrition) aged 18-30 years with persistent asthma that is not fully controlled (defined as a score of less than 19 on the ACT) [35]. We have intentionally chosen uncontrolled asthma patients as the program is designed to help take control of their asthma, and these patients would be the most motivated to use such an intervention. Participants must also own or have access to a cellular phone for the duration of the study and have a primary care physician. Recent data from the Pew Research Center demonstrate that 98% of Black individuals currently own a cellular phone, and that 96% of those aged 18-29 years own a smartphone [36]. To encourage participation, we will pay the cellular bill during the 8-week intervention period for all participants, a strategy that we and others have found to be extremely effective in this population. This will be in addition to the participation incentives. The exclusion criteria will be any other significant cardiopulmonary disease (including chronic obstructive pulmonary disease), a greater than 20 pack-year smoking history (as this level has been associated with the development of chronic obstructive pulmonary disease) [37], developmental delay or mental illness such that participation in the program would not be possible, and pregnancy. Women who become pregnant during the course of the study will be allowed to participate with written approval from their physician. Finally, as PAT will be part of the intervention, any subject who is unable to do mild physical activity for any health reason (including asthma) will be excluded. The ability to participate in mild physical activity will be verified by each participant’s primary care provider.

Participants will be recruited through clinical and community locations. Clinical locations will include the American Lung Association-Airway Clinical Research Centers network, as well as clinics at the University of Michigan, Wayne State University, and Detroit Medical Center. Community locations will utilize online recruitment primarily through social media, using procedures we have developed in previous work [38,39]. All components of the trial (including recruitment, signed informed consent, and intervention education components) will be done remotely.

### Proposed Intervention Components

#### MES

**Figure 3:**
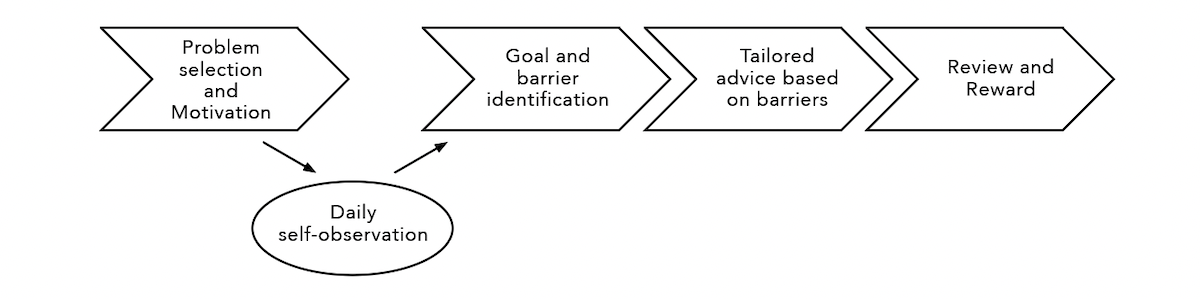
Flow of motivational enhancement system and supportive accountability intervention components.

The MES is a web-based mobile asthma management intervention delivered via the Computerized Intervention Authoring Software (CIAS 3.0) platform [40] on participants’ personal mobile devices. Despite being an automated mobile program, the MES is *tailored* for each participant in several ways. First, at baseline, participants will be classified according to their Asthma Self-Regulatory Development stage [41], which will guide behavioral content and number of sessions that each receives. Second, the MES is an interactive program that is individualized based on MI principles and participant-selected barriers/goals. Third, participants receive personalized feedback based on their responses to a daily diary completed between sessions 1 and 2, which prompts for barriers and facilitators to daily asthma management and control. Fourth, delivered content is based on a computer algorithm that incorporates the stage of asthma self-regulation to determine the number and frequency of sessions. Sessions are provided by an animated character (avatar) developed via focus groups with African Americans. Significant efforts have been made to ensure that the avatar delivers the intervention in a way that engages AAEAs with empathy, optimism, and autonomy support. The MES focuses on asthma management behaviors with feedback on asthma symptoms, physical activity, adherence, and tailored education. The avatar evokes both importance and confidence (key components of motivation) with MI strategies, such as identification of the benefits of behavior change, affirmations to reinforce change talk and boost confidence, and identification of personal strengths and resources. The avatar reflects (summarizes what the participant is saying to convey understanding) without judgment and provides statements to emphasize autonomy. Participants will follow a 4-step sequence of problem-solving based on the principles of the social cognitive theory, particularly self-regulation, and MI. This will include problem selection, self-observation with an online diary, goal selection, barrier selection, and reward selection, as participants progress through the 4 sessions (Figure 3).

#### SA

SA is an asthma management intervention delivered by asthma nurses trained in targeted MI skills (eg, open-ended questions around change talk and affirmations) via participants’ personal mobile devices (eg, Skype, FaceTime, voice calls, and SMS text messages). The theory underlying SA is that education and behavior change are most impactful when delivered by a knowledgeable yet supportive authority figure (ie, nurse) [42]. SA and the MES will be matched for number, timing, and focus of sessions. Sessions include promoting motivation for asthma management, education, feedback from participant reports of symptoms and management behaviors, enhancing self-efficacy, and goal setting. A total of 4 discrete sessions, as well as an asthma online diary, will be used in the SA intervention. SA sessions will also provide real-time exploration of barriers and problem-solving around asthma management, and will allow greater flexibility than the MES because they are delivered by a nurse interventionist versus an automated program. For instance, participants can choose topics beyond those on the MES list of most commonly chosen problems. SA calls include scripted components to check-in on asthma management activities but are otherwise flexible to allow focus on tailored barriers and issues a participant may have surrounding asthma management and control.

Asthma nurses will be provided with training in tailored MI. The following 3 provider communication behaviors have been found to be the strongest predictors of patient change talk: emphasizing patients’ autonomy, open-ended questions to elicit change talk, and reflections of change talk [43-45]. For this study, it is not feasible to train nurses to full MI competency, so our team has developed an approach to provide training in fundamental MI skills linked to behavior change [43]. Training will be provided by trainers certified by the Motivational Interviewing Network of Trainers and will include an initial 2-day workshop, followed by fidelity monitoring and boosters. MI fidelity will be monitored through audio recordings from SA sessions and rated using the MI Coach Rating Scale (MI-CRS) [46]. The MI-CRS is a 12-item measure of MI competence. Nurses will be trained in the interpretation of data from study-provided smart watches and will use this to encourage activity and asthma management.

#### TXT

SMS text messages will target asthma knowledge. These messages will be sent via CIAS 3.0 to participants’ personal mobile devices with facts about asthma management, links to educational web content, and videos providing information about living with asthma. A library of asthma education text messages developed during previous work will be refined and expanded using information from the CDC “Learn how to Control your Asthma” website [47]. Information covered includes “What is asthma,” “What is an asthma attack,” “What causes an asthma attack,” “How is asthma treated,” “Asthma and weather,” “Using an inhaler,” “Using a spacer,” and “Asthma action plans.” Messages will be sent twice a week for 4 weeks and then once a week for 4 weeks. SMS text messages will be formatted to fit a smart watch (see below) so that participants can use the study-provided device.

#### PAT

All participants, including those in the control arm, will be provided a smart watch in the form of an Apple Watch or Android watch. This PAT device will allow participants to accurately track exercise and total steps each day [48]. The US 2018 Physical Activity Guidelines Advisory Committee Scientific Report found “moderate” to “strong” evidence that mobile phones and wearable devices increase physical activity [49]. Through PAT, users will be able to set daily goals and attain or improve motivation to continue asthma management. For individuals randomized to groups that also include the MES or SA, those programs will incorporate PAT data that are remotely transmitted, to facilitate asthma management motivation. In formative work, AAEAs were enthusiastic regarding interventions that provided users a smart watch, and stated they were much more likely to continue in a trial that did so.

### Intervention Component Refinement

The MES, SA, and SMS components have been developed in previous work with different patient populations and behavioral targets. The current protocol includes 2 phases. During Phase 1 of the project, all 3 components will be refined and beta-tested for use with AAEAs having uncontrolled asthma. Minor adjustments will be required as we will now be integrating components both alone and in combination. Additionally, component refinement will be achieved with significant input from relevant stakeholders. Stakeholders include AAEAs with asthma, asthma nurses, asthma educators, primary care physicians, asthma specialty physicians, and computer specialists. All content has been developed through multi-step formative work, but it is important to continue to refresh and update content (eg, changing asthma guidelines and social phenomenon). For the primary stakeholder (AAEAs with asthma), 3 focus groups (4-6 individuals per group) will be held; the specifics of each component to be discussed are described below. Finally, prior to evaluation in the trial, beta-testing with a group of 10 AAEAs with asthma will be performed for each component arm to ensure that combinations are acceptable and well-integrated. Beta-testers will be asked to use assigned components, followed by an interview to obtain feedback on content, delivery, and technological issues.

### Qualitative Data Analysis

We will utilize a systematic, yet flexible, approach to thematic analysis to maintain rigor within the study timeline. Data will be managed and coded using NVivo 12. Two members of the research team will utilize the Framework Method, now used widely in health research [44-46,49-52]. Its defining feature is the matrix output, providing a structure to systematically reduce the data for analysis on various levels. After transcription, analysis will proceed through several stages: (1) familiarization with the session; (2) thematic coding; (3) developing a working analytical framework; (4) applying the framework; (5) charting data into the framework matrix; and (6) interpreting the data.

### Study Trial Design

This project will use the MOST [53-55] framework to optimize a mobile intervention to enhance the asthma management behaviors of AAEAs. The MOST design allows the evaluation of not only efficacy, but also efficiency, economy, scalability, and sustainability [56]. We will first refine the intervention components (MES, SA, PAT, and TXT) based on input from key stakeholders, including asthma nurses and AAEAs with asthma. We will then conduct a component selection experiment using a factorial research design to build an intervention that has been optimized for maximum efficacy. We will use a clinically significant improvement in asthma control (a change of ≥3 on the ACT [35]) as the criterion for determining which components should be kept in the optimized intervention. During the study, participants, their physicians, and any members of the study team who are adjudicators of outcomes will be unaware of study group assignment to the extent possible in a behavioral trial.

This study will use a 6-arm incomplete factorial design for the component selection experiment (Table 1). A full factorial design will not be used as some of the experimental combinations cannot be present simultaneously (eg, MES and SA are mHealth and involve human delivery of the same intervention content, and therefore logically cannot be combined), and PAT will be provided to all participants. Byar et al [57] described such incomplete designs, and in particular, we are employing “Design 3,” which is a “2^3^ − Y_AB_ − Y_ABC_” design. This design matches with our experimental combination as MES (A) and SA (B) components cannot occur together. Table 1 shows the incomplete factorial design given the possible combinations of the 4 components [55]. As the full factorial design is not feasible for the current setup, we also considered a 2^3−1^ fractional factorial design with 3 factors but with 4 (2^3−1^) treatment conditions, allowing aliasing of the highest-level interaction (ie, each main effect will be confounded with a 2-factor interaction). However, our design with 6 components is a superset of that 2^3−1^ fractional factorial design and will result in better evaluation of treatment combinations than any fractional factorial (see the study by Byar et al [57]). This design preserves much of the full factorial structure and provides more reasonable results than a fractional factorial design [58]. This design also allows evaluation of the main effect of each component and exploration of interaction effects when components are combined, though more assumptions are needed as a full factorial (=2^3^) is not present. One assumption is a “saturated design” when the combination missing (AB and ABC) is assumed to be 0 or have no effect. The component selection experiment is powered for the main effects and will include 180 participants (or 30 participants per arm).

**Table 1:** Intervention conditions.

Experimental condition	Motivational Enhancement System	SMS text messaging	Supportive accountability	Usual care	Physical activity tracking
1	Yes	No	No	No	Yes
2	No	Yes	No	No	Yes
3	No	No	Yes	No	Yes
4	Yes	Yes	No	No	Yes
5	No	Yes	Yes	No	Yes
6	No	No	No	Yes	Yes

### Study Procedures

All data collection and intervention activities will take place remotely. Following enrollment, research assistants will contact participants for consent and onboarding/orientation. Participants will receive US $25 per assessment for a total of US $100 and keep the Apple or Android watch. Participants will receive US $50 per month during the 2-month intervention period to offset the cost of their cell phone bill. To enhance engagement, this incentive will only be offered if participants remain above a determined threshold of response (eg, answer 80% of SA calls, text messages, etc), to be determined by key stakeholders during focus groups [59]. Data collection and most intervention activities will be conducted using CIAS 3.0. CIAS 3.0 is an HTML5 mobile web app with a responsive design capable of being deployed on any web browser accessed via any device (eg, Apple or Android smartphones) of any size (ie, auto reformat for optimal viewing on any size screen). This mobile version has an enhanced feature set, including improved voice quality for narrated content and appearance. Data collection sessions will take less than 30 minutes to complete. Participants will be randomized following baseline. Because the assessment is computer administered, there is no data collection staff per se for written measures.

#### Randomization

Participants will be randomized to 1 of 6 study arms following baseline. Randomization will be stratified by sex as we expect 65%-70% of subjects to be female (given the demographics of asthma among AAEAs). We will randomize using permuted blocks with sex as a stratification variable. Permuted blocks have the advantage of ensuring a balance between treatment arms for important prognostic variables without unmasking the next treatment allocation [60]. The randomization program/code will be developed and maintained centrally by a biostatistician.

#### Measurement

Data collection will occur at baseline, 3 months (postintervention), 6 months, and 12 months. At baseline, demographic information, asthma-specific information, and psychosocial information will be collected. The primary outcome will be improvement in asthma control as assessed by the ACT [35,61]. The mediators of asthma control will be based on the evaluation of IMB and SRT constructs, including asthma knowledge, attitudes, motivation, self-management behaviors, and self-efficacy.

Although we have feasibility data for the individual components, we will now obtain data for component combinations. Questionnaires and qualitative methodology will be used to determine recruitment and retention, adherence to the program, suitability and variability of outcome measures used, application and fidelity of the program, and demand/acceptability of the program for patients and professionals [62].

### Statistical Analysis

We will initially characterize the data heterogeneity and frequency distributions of asthma control, the primary outcome, and all secondary outcomes (asthma management and IMB/SRT constructs). We will check for out-of-range values, outliers, and abnormal values using graphical methods and create descriptive summaries to ensure that all values are within expected ranges. Unexpected findings will prompt checking of raw data for accuracy of data entry and recording. We will analyze the effect of the intervention components on the longitudinal measures of asthma control (ACT) using a mixed effects linear model for repeated measures analysis of variance (ANOVA) of a factorial design. This model will include a fixed effects indicator for each intervention component (SA, MES, TXT, and PAT) and time, along with all interactions with time. Random intercepts will be used to account for the longitudinal nature of the data. Before evaluating which components contribute to a potential change in the ACT score, we will use the model to compare the treatment with all 3 components and the control condition to determine whether the complete intervention is efficacious. If this statistical test is significant, we will identify those components that result in a significant change in the ACT score by examining the interactions between the main effects and time using the strategy advocated by Collins et al [53], which begins with the simplest effects and only adds higher-order interactions if needed. We will use a *P* value of <.05 for the test of total effect (difference between the treatment with all 3 components and the control treatment) and .1 to identify which components contribute to the total effect. We will use a higher threshold for the component selection test because we want to reduce the likelihood of not selecting a component that is contributing to the total effect. Secondary outcomes will be analyzed using a similar approach, but they are not powered. If significant baseline group imbalance is detected for any variable and its correlation with the outcome is 0.30, that variable will be included as a covariate in the inferential analyses. Dropouts and completers will also be compared in terms of baseline variables.

### Institutional Review Board Approval

The research protocol and study materials were reviewed and approved by the Wayne State University Social, Behavioral, and Education Review Board on September 1, 2021. Wayne State University (WSU) operates its Human Research Protection Program under a Federal wide Assurance (FWA) on file with the Office for Human Research Protection with identification number: FWA 00002460. The Social, Behavioral, Education board (B3) is IRB00000327.

## Results

Recruitment for intervention refinement via focus groups and beta-testing began in January 2022. Focus groups will be completed in March and April 2022. Recruitment for the trial is scheduled to begin in June 2022. The study results will be available within 12 months after the final data collection date (expected 2025). Results of Phase 1, including qualitative analyses from focus groups and the process of adaptation, will be available in late 2022. The findings will be disseminated to stakeholders using various methods, including peer-reviewed journals, academic conferences, and other communication modalities.

## Discussion

### Overview

Asthma causes substantial morbidity and mortality in the United States, particularly among AAEAs, but very few asthma programs have targeted this population and there are few interventions specifically designed to improve asthma control in this group. The study aims to significantly advance behavioral interventions for AAEAs with uncontrolled asthma. Interventions that provide education and address underlying motivation for managing asthma may be the most effective. However, intensive face-to-face interventions are often difficult to implement, especially among emerging adults. The purpose of this study is to develop an effective mobile asthma management intervention to improve asthma control among AAEAs. For this study, physical activity will be included as an important aspect of asthma management. The project utilizes the MOST framework to identify the best possible intervention component or combination of components to help AAEAs improve asthma control and, subsequently, lead healthier lives. At the completion of the study, we will have an empirically supported optimized mobile asthma management intervention to improve asthma control for AAEAs. Because the intervention is mobile and centralized, it has high potential for future scalability and implementation in clinical and community settings. Additionally, this project will enhance our understanding of barriers to and facilitators of asthma management and control among AAEAs, as well as the role of physical activity in managing asthma.

### Limitations

Although this study will lead to an optimized intervention for an underserved and at-risk population, there are potential limitations. Differential loss to follow-up is a threat to internal validity to assess the effect of components. Possible barriers to completion are high rates of no-shows at clinic visits and refusal to participate. Recruitment procedures and strategies have been the primary focus throughout our work because AAEAs with asthma are difficult to recruit. We have anticipated recruitment challenges and have developed community and online recruitment sources (social media). Another possible limitation is retaining participants for 12 months. Our research centers have an established history of working with at-risk urban populations and strong retention rates [38], and have developed various techniques to sustain engagement, including reminder letters, texts, and phone calls. Threats to the internal validity of the study may arise without sufficient attention to quality assurance of data collection and intervention delivery. Possible technological difficulties were minimal and remediated in previous work, resulting in protocols for technical support. Feasibility assessments and adaptive flexibility will be hallmarks of the protocol.

### Conclusions

This study will contribute to the existing literature on AAEAs with asthma; moreover, if successful, the project will result in an optimized intervention package specifically tailored for AAEAs with uncontrolled asthma to effectively improve asthma control. This is critical because emerging adulthood represents a unique period of development with challenges that may impact asthma management and asthma control; moreover, few studies and interventions have focused on improving asthma control among AAEAs. Planned next steps may include a large-scale multi-site effectiveness randomized controlled trial of the resulting optimized intervention package.

## Appendix

Multimedia Appendix 1 National Institutes of Health peer-review report.

## Abbreviations

AAEA: African American emerging adult
ACT: Asthma Control Test
CIAS: Computerized Intervention Authoring Software
CRS: Coach Rating Scale
IMB: Information-Motivation-Behavioral Skills
MES: Motivational Enhancement System
mHealth: mobile health
MI: motivational interviewing
MOST: multiphase optimization strategy
PAT: physical activity tracking
SA: supportive accountability
SRT: Self-Regulation Theory
TXT: SMS text messaging
