# Supplementary material for: Asthma and Technology in Emerging African American Adults (The ATHENA Project): Protocol for a Trial Using the Multiphase Optimization Strategy Framework
Source: JMIR Res Protoc. 2022 May 10;11(5):e37946. doi: 10.2196/37946 (PMC9131162; doi:10.2196/37946)
Supplement: Multimedia Appendix 1 [file resprot_v11i5e37946_app1.pdf]

SUMMARY STATEMENT

(Privileged Communication)

PROGRAM CONTACT:
MARTHA MATOCHA
301-594-2775
matocham@mail.nih.gov

Release Date: **02/22/2021**
Revised Date:

Application Number: **1 R01 NR019566-01A1**
Formerly: **1R01NR019566-01**

Principal Investigators (Listed Alphabetically):

BAPTIST, ALAN P.
MACDONELL, KAREN KOLMODIN (Contact)

Applicant Organization: WAYNE STATE UNIVERSITY

Review Group: **BMHO**
Biobehavioral Medicine and Health Outcomes Study Section

Meeting Date: **02/01/2021**
Council: **MAY 2021**
Requested Start: **07/01/2021**

RFA/PA: **PA18-386**
PCC: **AXZMM**
Dual IC(s): **EB**

Project Title: **Asthma and Technology in Emerging African American Adults (The ATHENA Project)**
SRG Action: **Impact Score:22 Percentile:10**
Next Steps: **Visit https://grants.nih.gov/grants/next_steps.htm**
Human Subjects: **48-At time of award, restrictions will apply**
Animal Subjects: **10-No live vertebrate animals involved for competing appl.**
Gender: **1A-Both genders, scientifically acceptable**
Minority: **2A-Only minorities, scientifically acceptable**
Age: **7A-Only Adults, scientifically acceptable**

Project Year	Direct Costs Requested	Estimated Total Cost
1	325,446	505,087
2	427,755	663,868
3	431,553	669,763
4	437,102	678,375
5	277,361	430,459
TOTAL	1,899,217	2,947,552

ADMINISTRATIVE BUDGET NOTE: The budget shown is the requested budget and has not been adjusted to reflect any recommendations made by reviewers. If an award is planned, the costs will be calculated by Institute grants management staff based on the recommendations outlined below in the COMMITTEE BUDGET RECOMMENDATIONS section.

1R01NR019566-01A1 MacDonell, Karen

PROTECTION OF HUMAN SUBJECTS UNACCEPTABLE

RESUME AND SUMMARY OF DISCUSSION: This application proposes to develop a mobile asthma management intervention to improve control in African American emerging adults (AAEA) and using a multiphase optimization strategy (MOST) framework will conduct a randomized six-arm incomplete factorial design experiment to identify which components or combinations of components effectively optimize asthma control. During the discussion, the panel agreed the significance of addressing health disparities in asthma management among AAEA through technology-based interventions informed by applied behavioral theories of change is high, with potential to advance asthma control in underserved populations. This strong multidisciplinary investigative team was responsive to prior review and the application is improved, notably by providing justification for the design, including preliminary data on feasibility and acceptability, and expanding the data analytic plan. Reviewers noted that using the MOST framework to evaluate intervention components and integrating two theoretical models are innovative approaches. Additional strengths include strong preliminary data, a methodology tailored to participants' goals and providing smartphones when needed. The panel also identified addressable weaknesses including that a comprehensive baseline assessment of asthma characteristics is not planned and the lack of discussion of sex disparities in asthma management, engagement in mobile health interventions and barriers to physical activity. Overall, the reviewers agreed the application's strengths outweighed its weaknesses and will have a high impact on advancing asthma management in African American emerging adults.

DESCRIPTION (provided by applicant): Asthma causes substantial morbidity and mortality in the U.S., particularly among African American emerging adults (AAEA; ages 18-30), but very few asthma programs have targeted this population. Interventions that provide education and address underlying motivation for managing asthma may be most effective. However, intensive, face-to-face interventions are often difficult to implement, especially among emerging adults. The purpose of this proposal is to develop an effective mobile asthma management intervention to improve control in AAEA. We will assess the ability of multiple technologic components to assist and improve traditional asthma education: 1) MES. The Motivational Enhancement System (MES) for Asthma Management is a mobile 4-session intervention utilizing supported self-regulation and Motivational Interviewing (MI). Personalized content is based on each participant's activity level, daily experiences, and goals. 2) SA. Supportive accountability (SA) is administered by asthma nurses utilizing targeted mobile support (Skype/voice calls) to provide education, promote self-efficacy, and overcome barriers through an MI-based framework. 3) SMS. Text messaging (SMS) provides reminders for asthma education, medication adherence, and physical activity. 4) PAT. Physical activity tracking (PAT) uses wearable technology to help meet user-defined physical activity goals. Using a multiphase optimization strategy (MOST) framework, we will test these 4 intervention components and combination of components to identify the most effective mobile intervention. MOST is an innovative, cost- and time-effective framework that utilizes engineering principles to produce effective behavioral interventions. We will conduct a component selection experiment using a factorial research design to build an intervention that has been optimized for maximum efficacy. We will use a clinically significant improvement in asthma control as the criterion for determining which components should be kept in the optimized intervention. Participants (N=180) will be randomized to 1 of 6 intervention arms consisting of various combinations of the intervention components. This experimental design is equivalent to conducting multiple pilot randomized clinical trials to evaluate the efficacy of each component, yet uses only a fraction of the sample size and resources. At the completion of the study, we will have an empirically-supported, optimized mobile asthma management intervention to improve asthma control for AAEA. Participants will be recruited from multiple sites of the American Lung Association Airway Clinical

Research Center network and ambulatory care clinics at the Detroit Medical Center. Data collections will occur at baseline, 3, 6, and 12 months. We hypothesize that post-intervention (3, 6, and 12 months), participants with uncontrolled asthma will show clinically-significant improvement in asthma control. We hypothesize that improvements in asthma management behaviors (including physical activity), quality of life, symptoms, adherence, and exacerbations (secondary outcomes) will also be observed.

PUBLIC HEALTH RELEVANCE: African American emerging adults (ages 18-30) are disproportionately impacted by asthma, but have been under-represented in research. Mobile asthma management interventions may help improve asthma control and allow people to live healthier lives. During this project, we will use an innovative strategy to develop an optimized mobile asthma management intervention using the most effective combination of nurse-delivered asthma education, a smart phone app, and text messaging.

CRITIQUE 1

Significance: 2
Investigator(s): 2
Innovation: 4
Approach: 3
Environment: 2

Overall Impact: In this R01 resubmission, the investigators propose a randomized trial with MOST design to evaluate the effects of different mHealth interventions to improve asthma control among African American emerging adults with uncontrolled asthma. The application has multiple important strengths. First, it is highly significant because of its focus on an underserved population of young adults that accounts for a substantial proportion of asthma morbidity. Second, the scientific premise of the application is built upon extensive work by the co-PIs that have applied behavioral theories of change to develop effective technology-based interventions to improve asthma outcome sin underserved populations. Overall, the investigative team and intellectual environment are strong and provide all elements necessary for the successful completion of the proposed studies. Although the application is essentially the continuation of ongoing work by the investigators and it is not particularly novel, the proposed clinical trial represents the first study that would to test different components individually and in combination in a sufficient number of participants and with a follow-up period of up to one year. Overall, the study is scientifically rigorous, possible pitfalls are addressed, and multiple outcomes evaluated within a rigorous study design. Among the weaknesses are the lack of consideration for possible effects of other asthma-related factors (including current treatment, health care access, obesity, allergy to specific allergens), the lack of a comprehensive baseline assessment of asthma clinical characteristics, and the inclusion of possibly heavy smokers that may introduce an independent source of obstructive lung disease (other than asthma). Overall, strengths outweigh weaknesses and this application is likely to have a significant impact in the field of asthma managements among AAEAs.

1. Significance:

Strengths

- The scientific premise of this application is strong and based on previous work that this investigative team (and the two co-PIs in particular) have completed in using technology to

improve asthma management in African Americans. The proposed MOST trial is the continuation of this work

- The focus on African Americans (as an underserved population) and on emerging adults (as a phase of life particularly susceptible to negative outcomes in asthmatics) reinforces the significance of the proposed study
- The significance of some elements of the design (e.g., completion of all trial phases remotely, systematic use of cell phones and other electronic devices in asthma management) is also enhanced in the context of the COVID pandemic

Weaknesses

- None noted

2. Investigator(s):

Strengths

- Dr MacDonell is an associate professor with extensive experience in the use of technology to improve asthma management in African Americans. She is the PI of an ongoing RCT to test a multi-component technology-based intervention in AAs with asthma, which has provided critical preliminary data and scientific rationale in support of the current application
- Dr Baptist (co-PI) is the Director of the U of Michigan Asthma program and a clinician with an established interest in applying health education to overcome disparities in asthma. The two co-PIs provide strong and complementary expertise in the context of the proposed studies
- The investigative team is excellent and provides the diverse expertise necessary to complete all elements of the proposed trial successfully

Weaknesses

- No history of previous collaborations between the two co-PIs
- Because Dr Naar will play a central role in some components of the study, a biosketch should have been provided

3. Innovation:

Strengths

- Although none of the proposed components is truly novel, their application in the context of AAEAs and of the MOST study design is innovative
- This will be the first trail to test different components individually and in combination in a sufficient number of participants and with a follow-up period of up to one year

Weaknesses

- Much of this work appears to be an expansion of previous clinical trials completed by this group

4. Approach:

Strengths

- The scientific rationale of the study is solidly based in behavioral theories of change and the effectiveness of technology-based interventions

- The MOST design will allow the evaluation of multiple components (MES, SA, SMS, PAT) individually and in combination in a highly cost-effective fashion
- Procedures for refinement and beta-testing are well-integrated in the design
- Overall, the study is scientifically rigorous, possible pitfalls are addressed, and multiple outcomes evaluated within a rigorous study design

Weaknesses

- The possible effects of baseline asthma characteristics (including current treatment, health care access, obesity, allergy to specific allergens) are not considered.
- The lack of a comprehensive baseline assessment of asthma characteristics (including clinical elements, such as lung function and FeNO) may also limit the conclusions that will be reached from this trial
- Including smokers with up to 20 pack-years may introduce an independent source of obstructive lung disease (other than asthma)

5. Environment:

Strengths

- Excellent environment. Previous work by this group has been completed at Wayne State University. Clinical centers at the University of Michigan and within the ALA-ACRC network will provide excellent settings for patient recruitment

Weaknesses

- None noted

Study Timeline:

Strengths

- Training, component refinement, and beta-testing have been all factored in the timeline
- Recruitment goals appear achievable

Weaknesses

- Recruitment is planned well into year 4, which may push completion of the 1-year follow-up for some participants quite close to the end of the project

Protections for Human Subjects:

Unacceptable Risks and/or Inadequate Protections

- A response by the care provider should be required in order for the patient to participate in the study

Data and Safety Monitoring Plan (Applicable for Clinical Trials Only):

Acceptable

Inclusion Plans:

- Sex/Gender: Distribution justified scientifically

- Race/Ethnicity: Distribution justified scientifically
- Inclusion/Exclusion Based on Age: Distribution justified scientifically

Vertebrate Animals:

Not Applicable (No Vertebrate Animals)

Biohazards:

Not Applicable (No Biohazards)

Resubmission:

- investigators have been largely responsive to previous critiques. Most notably, the duration of follow-up has been extended to one year, the payment model has been revised, preliminary data from the ongoing RCT have been integrated in the design, and the description of the MOST study design and statistical approach has been expanded and improved.

Resource Sharing Plans:

Acceptable

Budget and Period of Support:

Recommend as Requested

CRITIQUE 2

Significance: 3
Investigator(s): 2
Innovation: 3
Approach: 3
Environment: 2

Overall Impact: This application seeks to refine and test an intervention to improve asthma control among young African Americans with asthma. The resubmission is responsive to prior critiques, revising the participant compensation model, follow-up period, statistical analysis plan, and clarifying innovative aspects. The need for refinement of the intervention is not a major weakness and fits well within the study timeline. Strengths include a focus on improving health outcomes among African Americans, the rigorous randomized MOST trial design, and the strong study team. Weaknesses include limited justification for using smart watches and overlooking sex as a potentially important factor given the potential sex differences in barriers to increasing physical activity and engaging with digital health interventions. Overall, strengths outweigh these weaknesses, and the project is expected to have high impact.

1. Significance:

Strengths

- Improving outcomes among individuals with asthma, a chronic and debilitating disease, will have significant public health impact.
- This application is based on rigorous prior research of the intervention components, which enhances the significance of this project.

Weaknesses

- Sex is overlooked as a potentially important variable in this mostly female population.

2. Investigator(s):

Strengths

- The investigative team is strong and well suited to this project.
- The study team has the requisite expertise and training to successfully carry out the project.

Weaknesses

- None noted.

3. Innovation:

Strengths

- Integrating two theoretical approaches and multiple efficacious interventions will shift research and practice paradigms towards these approaches.
- Targeting young African Americans with asthma is innovative.
- The multiphase optimization strategy (MOST) design is innovative and efficient.

Weaknesses

- None noted.

4. Approach:

Strengths

- Use of a MOST design enhances the likelihood of the project's success in accomplishing the proposed aims.
- The statistical analysis plan and sample size justification are thorough and appropriate to the project.

Weaknesses

- Use of a smartwatch to track physical activity over less expensive options (i.e., fitness trackers) is not well justified and limits the potential uptake of this approach.

5. Environment:

Strengths

- The study environment is excellent and well suited to the project.

Weaknesses

- None noted.

Study Timeline:

Strengths

- The study timeline is acceptable.

Weaknesses

- None noted.

Protections for Human Subjects:

Acceptable Risks and/or Adequate Protections

- Acceptable risks and adequate protections are described.

Data and Safety Monitoring Plan (Applicable for Clinical Trials Only):

Acceptable

- The data and safety monitoring plan is acceptable.

Inclusion Plans:

- Sex/Gender: Distribution justified scientifically
- Race/Ethnicity: Distribution justified scientifically
- Inclusion/Exclusion Based on Age: Distribution justified scientifically
- Inclusion plans are acceptable.

Vertebrate Animals:

Not Applicable (No Vertebrate Animals)

Biohazards:

Not Applicable (No Biohazards)

Resubmission:

- The resubmission is responsive to prior critiques. The participant compensation model has been revised, the follow-up period has been extended, the statistical analysis plan has been revised, and innovative aspects of the project have been clarified. The need for refinement of the intervention is not a major weakness and fits well within the study timeline.

Resource Sharing Plans:

Acceptable

Budget and Period of Support:

Recommend as Requested

CRITIQUE 3

Significance: 3
Investigator(s): 2
Innovation: 3
Approach: 4
Environment: 2

Overall Impact: The application is a resubmission of a previous R01 application. The proposed project addresses the significant health disparities issue with respect to asthma management among African American emerging adults (AAEA). The proposed project applies an innovative multiphase optimal strategy (MOST) framework to identify which components or combinations of components are most efficacious and efficient in improving asthma control and associated secondary outcomes (quality of life, asthma exacerbations, physical activity). The environment and investigative team are strong with demonstrated expertise in asthma behavioral interventions and mHealth and technological approaches within the target population. Weaknesses of the application include its limited generalizability due to the narrow focus on AAEA with asthma while ignoring other groups which face significant asthma disparities, lack of consideration of demographic characteristics or health comorbidities which may influence both intervention engagement and asthma management. The study team was responsive to previous critiques and made considerable changes to strengthen the application. Among the most important changes were the justification for the incomplete factorial design within the MOST framework, greater clarity regarding refinement of intervention components, inclusion of preliminary data on study components' feasibility and acceptability, and monetary considerations around cell phone access for participants through the intervention duration.

1. Significance:

Strengths

- The application is significant in its focus on reducing asthma disparities among African American emerging adults (AAEA).
- Interventions to improve asthma self-management among AAEA are greatly needed, as this is a population that is under-researched in this field.
- Asthma disparities among AA compared to non-Hispanic whites persist, with AA having a higher lifetime prevalence of asthma and nearly a 3-fold higher mortality rate.

Weaknesses

- Generalizability of the intervention is limited by the narrow focus on AAEA ages 18-39. Other groups, including Latino (in particular Puerto Rican) EA also experience significant asthma disparities, however these are not acknowledged.

2. Investigator(s):

Strengths

- MPI team of Drs. MacDonnell and Baptist bring complimentary expertise in the fields of health psychology and medicine respectively.
- The PIs individually and collectively have a history of scholarship and funding in the areas of asthma management.

- The study team is well suited to carry out the proposed study with additional expertise in biostatistics, mHealth, behavioral interventions (including MOST and SMART designs), nursing, and social work.

Weaknesses

- None

3. Innovation:

Strengths

- Key area of innovation is the use of the MOST design which is under-utilized among interventions to reduce asthma disparities.

Weaknesses

- Neither motivational interviewing-based interventions nor mHealth based approaches to asthma management are particularly innovative.

4. Approach:

Strengths

- The MOST framework is an efficient design to assess which component(s) are most efficacious in improving asthma outcomes within this population. While the study proposes to use an incomplete factorial design, the reasons for this are well justified.
- Intervention components are theoretically grounded (MI and SRT) and tailored to participants' needs, goals, and preferences.
- The primary outcomes are clinically meaningful and the increase in length of follow-up to 1 year provides opportunity to observe sustained changes in behavioral management.
- Preliminary data regarding feasibility and acceptability of intervention components, retention of participants is included.
- Letters of support from additional American Lung Association – Airway Clinical Research Centers network sites are included.

Weaknesses

- Randomization by sex is mentioned, given that mPIs anticipate 65-70% of participants to be female, but there is no prior mention of additional tailoring of intervention components by sex. This is a concern given the existing literature regarding sex disparities in asthma management, engagement with mHealth and technological interventions, and barriers to physical activity.
- There is no mention of demographic or psychosocial factors (e.g. mental health comorbidities) which may impact both engagement with the intervention and also asthma control.
- No dissemination plan is included which seems like a missed opportunity given the engagement of various stakeholders in both the recruitment and refinement phases of the proposed study.

5. Environment:

Strengths

- Resources from the University of Michigan and Wayne State University, as well as additional recruitment from other ALA-ACR sites, are appropriate to conduct the proposed studies.

Weaknesses

- None

Study Timeline:

Strengths

- Adequate time for recruitment of study participants
- Adequate follow up of 1 year for study participants.

Weaknesses

- None

Protections for Human Subjects:

Acceptable Risks and/or Adequate Protections

- Protections of human subjects against risk appear adequate.

Data and Safety Monitoring Plan (Applicable for Clinical Trials Only):

Acceptable

- Includes establishment of DSMB; DSMP is appropriate for the scope of the study.

Inclusion Plans:

- Sex/Gender: Distribution justified scientifically
- Race/Ethnicity: Distribution justified scientifically
- Inclusion/Exclusion Based on Age: Distribution justified scientifically
- Hispanic and Latino participants are included in the enrollment numbers, but no within the study. Participants include African American emerging adults ages 18 years to 29 years.

Vertebrate Animals:

Not Applicable (No Vertebrate Animals)

Biohazards:

Not Applicable (No Biohazards)

Resubmission:

- Overall, study team was responsive to previous critiques and made considerable changes to strengthen the application. Among the most important changes were the justification for the incomplete factorial design within the MOST framework, greater clarity regarding refinement of intervention components, inclusion of preliminary data on study components' feasibility and acceptability, and monetary considerations around cell phone access for participants through the intervention duration.

Resource Sharing Plans:

Not Applicable (No Relevant Resources)

Budget and Period of Support:

Recommend as Requested

THE FOLLOWING SECTIONS WERE PREPARED BY THE SCIENTIFIC REVIEW OFFICER TO SUMMARIZE THE OUTCOME OF DISCUSSIONS OF THE REVIEW COMMITTEE, OR REVIEWERS' WRITTEN CRITIQUES, ON THE FOLLOWING ISSUES:

PROTECTION OF HUMAN SUBJECTS: UNACCEPTABLE

The panel agreed that physical activity in uncontrolled asthma can trigger an attack, so not hearing back from a participant's physician should not be considered as approval to participate in the study.

INCLUSION OF WOMEN PLAN: ACCEPTABLE

INCLUSION OF MINORITIES PLAN: ACCEPTABLE

INCLUSION ACROSS THE LIFESPAN: ACCEPTABLE

COMMITTEE BUDGET RECOMMENDATIONS: The budget was recommended as requested.

Footnotes for 1 R01 NR019566-01A1; PI Name: MacDonell, Karen Kolmodin

NIH has modified its policy regarding the receipt of resubmissions (amended applications).See Guide Notice NOT-OD-18-197 at https://grants.nih.gov/grants/guide/notice-files/NOT-OD-18-197.html. The impact/priority score is calculated after discussion of an application by averaging the overall scores (1-9) given by all voting reviewers on the committee and multiplying by 10. The criterion scores are submitted prior to the meeting by the individual reviewers assigned to an application, and are not discussed specifically at the review meeting or calculated into the overall impact score. Some applications also receive a percentile ranking. For details on the review process, see http://grants.nih.gov/grants/peer_review_process.htm#scoring.

MEETING ROSTER

Biobehavioral Medicine and Health Outcomes Study Section
Risk, Prevention and Health Behavior Integrated Review Group
CENTER FOR SCIENTIFIC REVIEW
BMHO
02/01/2021 - 02/02/2021

Notice of NIH Policy to All Applicants: Meeting rosters are provided for information purposes only. Applicant investigators and institutional officials must not communicate directly with study section members about an application before or after the review. Failure to observe this policy will create a serious breach of integrity in the peer review process, and may lead to actions outlined in NOT-OD-14-073 at https://grants.nih.gov/grants/guide/notice-files/NOT-OD-14-073.html and NOT-OD-15-106 at https://grants.nih.gov/grants/guide/notice-files/NOT-OD-15-106.html, including removal of the application from immediate review.

CHAIRPERSON(S)

FILLINGIM, ROGER B, PHD
DISTINGUISHED PROFESSOR
DEPARTMENT OF COMMUNITY DENTISTRY
AND BEHAVIORAL SCIENCE
COLLEGE OF DENTISTRY
UNIVERSITY OF FLORIDA
GAINESVILLE, FL 32610

MEMBERS

BRUEHL, STEPHEN, PHD
PROFESSOR
DEPARTMENT OF ANESTHESIOLOGY
VANDERBILT UNIVERSITY MEDICAL CENTER
NASHVILLE, TN 37212

BURG, MATTHEW M, PHD
PROFESSOR
DEPARTMENT OF INTERNAL MEDICINE
YALE UNIVERSITY SCHOOL OF MEDICINE
NEW HAVEN, CT 06520

BUTLER, ASHLEY M, PHD *
ASSOCIATE PROFESSOR
DEPARTMENT OF PEDIATRICS
BAYLOR COLLEGE OF MEDICINE
HOUSTON, TX 77030

CAMACHO, MARLENE, DSC, MPH *
ASSISTANT PROFESSOR
DEPARTMENT OF COMMUNITY HEALTH SERVICES
SUNY DOWNSTATE HEALTH SCIENCES UNIVERSITY
BROOKLYN, NY 11203

CASTLE, JESSICA R, MD *
ASSOCIATE PROFESSOR
DEPARTMENT OF MEDICINE
OREGON HEALTH AND SCIENCE UNIVERSITY
PORTLAND, OR 97219

CHAYTOR, NAOMI S, PHD *
ASSOCIATE PROFESSOR
DEPARTMENT OF MEDICAL EDUCATION
AND CLINICAL SCIENCES
ELSON S. FLOYD COLLEGE OF MEDICINE
WASHINGTON STATE UNIVERSITY
SPOKANE, WA 99202

COLLOCA, LUANA, PHD *
ASSOCIATE PROFESSOR
DEPARTMENT OF PAIN TRANSLATIONAL SYMPTOM
SCIENCE
SCHOOL OF NURSING
UNIVERSITY OF MARYLAND
BALTIMORE, MD 21201

FORTIER, MICHELLE, PHD
ASSOCIATE PROFESSOR
SUE AND BILL GROSS SCHOOL OF NURSING
UNIVERSITY OF CALIFORNIA, IRVINE
ORANGE, CA 92868

GALLO, LINDA C, PHD *
PROFESSOR
DEPARTMENT OF PSYCHOLOGY
SAN DIEGO STATE UNIVERSITY
SAN DIEGO, CA 92123

GOLDBERG, DAVID S, MD, MS *
ASSOCIATE PROFESSOR
DIVISION OF DIGESTIVE HEALTH AND LIVER
DEPARTMENT OF MEDICINE
UNIVERSITY OF MIAMI MILLER SCHOOL OF MEDICINE
MIAMI, FL 33136

GONZALEZ, BRIAN D, PHD *
ASSISTANT MEMBER AND SCIENTIFIC DIRECTOR
MOFFITT CANCER CENTER
MAGNOLIA CAMPUS
TAMPA, FL 33647

GUERRA, STEFANO, PHD *
PROFESSOR
ARIZONA RESPIRATORY CENTER
UNIVERSITY OF ARIZONA
TUCSON, AZ 85724

HARPER, FELICITY, PHD
ASSOCIATE PROFESSOR
DEPARTMENT OF ONCOLOGY
KARMANOS CANCER INSTITUTE
WAYNE STATE UNIVERSITY
DETROIT, MI 48201

HARVEY, JEAN R, PHD
PROFESSOR
DEPARTMENT OF NUTRITION AND FOOD SCIENCES
UNIVERSITY OF VERMONT
BURLINGTON, VT 05405

HASSETT, AFTON L, PSYD
ASSOCIATE PROFESSOR
DEPARTMENT OF ANESTHESIOLOGY
CHRONIC PAIN AND FATIGUE RESEARCH CENTER
UNIVERSITY OF MICHIGAN MEDICAL SCHOOL
ANN ARBOR, MI 48106

KRONISH, IAN M, MD
ASSOCIATE PROFESSOR
DEPARTMENT OF MEDICINE
COLUMBIA UNIVERSITY MEDICAL CENTER
NEW YORK, NY 10032

LABUS, JENNIFER S, PHD *
PROFESSOR
CENTER FOR NEUROBIOLOGY OF STRESS AND RESILIENCE
LOS ANGELES, CA 90095

LONG, CHRISTOPHER, PHD *
ASSISTANT PROFESSOR
DEPARTMENT OF PSYCHIATRY
UNIVERSITY OF ARKANSAS FOR MEDICAL SCIENCES
FAYETTEVILLE, AR 72703

MARTIN, MOLLY A, MD *
ASSOCIATE PROFESSOR
DEPARTMENT OF PEDIATRICS
INSTITUTE FOR HEALTH RESEARCH AND POLICY
UNIVERSITY OF ILLINOIS AT CHICAGO
CHICAGO , IL 60612

MATHEW, JOSEPH P, MD, MHSC
PROFESSOR
DEPARTMENT OF ANESTHESIOLOGY
DUKE UNIVERSITY MEDICAL CENTER
DURHAM, NC 27710

MERLIN, JESSICA S, MD *
ASSOCIATE PROFESSOR
DIVISION OF GENERAL INTERNAL MEDICINE ACADEMIC OFFICES
UPMC MONTEFIORE HOSPITAL
PITTSBURGH, MD 15213

NAPADOW, VITALY, PHD *
ASSOCIATE PROFESSOR
MARTINOS CENTER FOR BIOMEDICAL IMAGING
MASSACHUSETTS GENERAL HOSPITAL
HARVARD MEDICAL SCHOOL
CHARLESTOWN, MA 02129

NEELON, SARA E, PHD
PROFESSOR
DEPARTMENT OF HEALTH
BEHAVIOR AND SOCIETY
JOHNS HOPKINS SCHOOL OF PUBLIC HEALTH
BALTIMORE, MD 21205

RADHAKRISHNAN, KAVITA, PHD *
ASSOCIATE PROFESSOR
SCHOOL OF NURSING
THE UNIVERSITY OF TEXAS - AUSTIN
AUSTIN, TX 78701

RHEE, KYUNG E, MD
PROFESSOR
DEPARTMENT OF PEDIATRICS
UNIVERSITY OF CALIFORNIA, SAN DIEGO
LA JOLLA, CA 92093

RINI, CHRISTINE, PHD
PROFESSOR
DEPARTMENT OF MEDICAL SOCIAL SCIENCES
NORTHWESTERN UNIVERSITY
FEINBERG SCHOOL OF MEDICINE
CHICAGO, IL 60611

ROGERS, CHARLES R, PHD, MPH *
ASSISTANT PROFESSOR
FAMILY & PREVENTIVE MEDICINE
UNIVERSITY OF UTAH SCHOOL OF MEDICINE
SALT LAKE CITY, UT 84108

ROPER, JENEVIEVE, PHD *
ASSISTANT PROFESSOR
HEALTH AND HUMAN SCIENCES
LOYOLA MARYMOUNT UNIVERSITY
LOS ANGELES, CA 90045

SHIN, ANDREA, MD, CLIN SCI *
ASSISTANT PROFESSOR
DEPARTMENT OF MEDICINE
INDIANA UNIVERSITY
INDIANAPOLIS, IN 46202

SMITH, PATRICK JOSEY, PHD, MPH
ASSOCIATE PROFESSOR
DEPARTMENT OF PSYCHIATRY AND BEHAVIORAL SCIENCES
MEDICINE AND POPULATION HEALTH SCIENCES
DUKE UNIVERSITY
DURHAM, NC 27710

SMITH, SAKIMA AHMAD, MD *
ASSOCIATE PROFESSOR
DIVISION OF CARDIOVASCULAR MEDICINE
DEPARTMENT OF INTERNAL MEDICINE
WEXNER MEDICAL CENTER
THE OHIO STATE UNIVERSITY
COLUMBUS, OH 43210

TEMPLE, JENNIFER L, PHD
PROFESSOR
DEPARTMENTS OF EXERCISE AND NUTRITION SCIENCES AND COMMUNITY HEALTH AND HEALTH BEHAVIOR
SCHOOL OF PUBLIC HEALTH AND HEALTH PROFESSIONS
UNIVERSITY AT BUFFALO
BUFFALO, NY 14214

UEBELACKER, LISA A, PHD
PROFESSOR
DEPARTMENT OF PSYCHIATRY AND HUMAN BEHAVIOR
BUTLER HOSPITAL
BROWN UNIVERSITY
PROVIDENCE, RI 02906

WEN, KUANG-YI, PHD *
ASSOCIATE PROFESSOR
THOMAS JEFFERSON UNIVERSITY
SIDNEY KIMMEL CANCER CENTER
PHILADELPHIA, PA 19107

MAIL REVIEWER(S)

REYLAND, MARY E, PHD
DIRECTOR, GRADUATE PROGRAM IN CANCER BIOLOGY, PROFESSOR WITH TENURE
DEPARTMENT OF CRANIOFACIAL BIOLOGY
SCHOOL OF DENTAL MEDICINE
ANSHUTZ MEDICAL CAMPUS
UNIVERSITY OF COLORADO, DENVER
AURORA, CO 80045

SCIENTIFIC REVIEW OFFICER

VOSVICK, MARK A, PHD
SCIENTIFIC REVIEW OFFICER
CENTER FOR SCIENTIFIC REVIEW
NATIONAL INSTITUTES OF HEALTH
BETHESDA, MD 20892

EXTRAMURAL SUPPORT ASSISTANT

WATTS, MELISSA D
EXTRAMURAL SUPPORT ASSISTANT
CENTER FOR SCIENTIFIC REVIEW
NATIONAL INSTITUTE FOR HEALTH
BETHESDA, MD 20892

* Temporary Member. For grant applications, temporary members may participate in the entire meeting or may review only selected applications as needed.

Consultants are required to absent themselves from the room during the review of any application if their presence would constitute or appear to constitute a conflict of interest.
